# Plasma Fatty Acids in Zambian Adults with HIV/AIDS: Relation to Dietary Intake and Cardiovascular Risk Factors

**DOI:** 10.1155/2015/635817

**Published:** 2015-05-26

**Authors:** Christopher K. Nyirenda, Edmond K. Kabagambe, John R. Koethe, James N. Kiage, Benjamin H. Chi, Patrick Musonda, Meridith Blevins, Claire N. Bosire, Michael Y. Tsai, Douglas C. Heimburger

**Affiliations:** ^1^Ndola Central Hospital, School of Medicine, 10101 Ndola, Zambia; ^2^School of Medicine, Copperbelt University, 10101 Ndola, Zambia; ^3^Vanderbilt Institute for Global Health, Vanderbilt University, Nashville, TN 37203, USA; ^4^Division of Epidemiology, Department of Medicine, Vanderbilt University Medical Center, Nashville, TN 37203, USA; ^5^Division of Infectious Diseases, Department of Medicine, Vanderbilt University Medical Center, Nashville, TN 37232, USA; ^6^Centre for Infectious Disease Research in Zambia, 10101 Lusaka, Zambia; ^7^Department of Obstetrics and Gynecology, University of North Carolina at Chapel Hill, Chapel Hill, NC 27599, USA; ^8^Department of Biostatistics, Vanderbilt University Medical Center, Nashville, TN 37203, USA; ^9^Division of Cancer Epidemiology and Genetics, National Cancer Institute, Nutritional Epidemiology Branch, Bethesda, MD 20850, USA; ^10^Department of Laboratory Medicine and Pathology, University of Minnesota Medical School, Minneapolis, MN 55455, USA

## Abstract

*Objective*. To determine whether 24 hr dietary recalls (DR) are a good measure of polyunsaturated fatty acid (PUFA) intake when compared to plasma levels, and whether plasma PUFA is associated with markers of HIV/AIDS progression and cardiovascular disease (CVD) risk. *Methods*. In a cross-sectional study among 210 antiretroviral therapy-naïve HIV-infected adults from Lusaka, Zambia, we collected data on medical history and dietary intake using 24 hr DR. We measured fatty acids and markers of AIDS progression and CVD risk in fasting plasma collected at baseline. *Results*. PUFA intakes showed modest correlations with corresponding plasma levels; Spearman correlations were 0.36 (*p* < 0.01) for eicosapentaenoic acid and 0.21 (*p* = 0.005) for docosahexaenoic acid. While there were no significant associations (*p* > 0.05) between total plasma PUFA and C-reactive protein (CRP) or lipid levels, plasma arachidonic acid was inversely associated with CRP and triglycerides and positively associated with HDL-C, CD4+ T-cell count, and plasma albumin (*p* < 0.05). Plasma saturated fatty acids (SFA) were positively associated with CRP (*β* = 0.24; 95% CI: 0.08 to 0.40, *p* = 0.003) and triglycerides (*β* = 0.08; 95% CI: 0.03 to 0.12, *p* < 0.01). *Conclusions*. Our data suggest that a single DR is inadequate for assessing PUFA intake and that plasma arachidonic acid levels may modulate HIV/AIDS progression and CVD risk.

## 1. Introduction

HIV/AIDS is a major cause of morbidity and mortality in Zambia, where in 2009 the national HIV prevalence in the 15–49 year age group was estimated at 13.5% [1], but effective treatment is complicated by a high degree of malnutrition in the HIV-infected population. A review of HIV-infected adults starting antiretroviral therapy (ART) in Lusaka, Zambia, found 33% of patients were undernourished by World Health Organization criteria (e.g., a body mass index (BMI) < 18.5 kg/m^2^), and 9% were severely malnourished (BMI < 16 kg/m^2^) [2]. Mortality in the early ART treatment period is higher among patients with low BMI, low CD4+ T-cell counts, low hemoglobin, low serum albumin and phosphate levels, advanced HIV/AIDS stage, and immune reconstitution inflammatory syndrome [3–5].

In Zambia, our group recently reported a higher risk of early ART mortality among individuals with moderately elevated triglyceride concentrations, a finding which may be related to the interaction of fatty acids and systemic inflammation [6]. The essential fatty acids (n-3 and n-6 polyunsaturated fatty acids (PUFA)) are nutrients of primary importance for health, and many research works in the last decades have shown the role of an adequate intake of n-3 and n-6 PUFA in the prevention of several diseases, in particular of cardiovascular diseases [7–9]. These studies were done mainly in non-HIV populations. Omega 3 fatty acids have been known to modulate biomarkers such as C-reactive protein (CRP) and CD4 count which are important determinants in the progression of HIV disease and other inflammatory conditions [10, 11]. To our knowledge, apart from one small clinical trial that assessed the benefit of combining fenofibrate with n-3 fatty acids in improving HIV-related clinical outcomes [12], studies on effects of fatty acids on metabolic parameters associated with morbidity and mortality in HIV are lacking, especially in resource-limited settings.

Reliable identification of individuals with key nutrient deficiencies that could be improved with nutritional support would inform the design of nutritional rehabilitation programs to reduce morbidity and mortality in HIV/AIDS patients. The Zambian diet is mainly composed of cereals, predominantly maize, starchy roots, and, to a lesser extent, fruits and vegetables. Cereals provide almost two-thirds of the dietary energy supply.

In urban areas of Zambia food consumption patterns are changing: rice and sweet potatoes are gaining importance [13]. Urbanization and globalization are responsible for changes in dietary patterns, as consumption is shifting from fresh and minimally processed traditional foods to imported processed foods acquired from supermarkets [14]. Because fish intake in much of Zambia is relatively low [15], we hypothesized that dietary intake of long-chain PUFA may be inadequate in HIV-infected adults and could influence markers of CVD risk and HIV disease progression. In this study, we sought to determine whether 24-hr dietary recalls (DR) conducted in an urban ART clinic setting in Zambia could be used to effectively estimate PUFA intake and whether PUFA and saturated fatty acids (SFA) measured in plasma are associated with markers of cardiovascular disease risk.

## 2. Methods

### 2.1. Study Population

We enrolled HIV-infected adults (age 16–60 years) eligible for ART initiation into the diet, genetic polymorphisms in lipid-metabolizing enzyme genes, and antiretroviral therapy-related dyslipidemia (DGPLEAD) study at Chawama Clinic, a government health centre in Lusaka, Zambia, between January and December 2007. All participants had a BMI ≥16 kg/m^2^ and CD4+ lymphocyte count ≥50 cells/*μ*L, and were ART eligible according to the Zambia national HIV guidelines. The DGPLEAD study has been described in detail previously [6]. Dietary and metabolic profiles described in this study were assessed before initiating ART. Written informed consent was obtained from all participants during the primary study. The research protocol was approved by the Vanderbilt University Institutional Review Board in Nashville Tennessee, USA, and the University of Zambia Biomedical Research Ethics Committee in Lusaka, Zambia.

### 2.2. Data Collection

Data collection was conducted by a study nurse, a clinical officer, and a supervising physician (CKN). At the first encounter, medical history and physical examination with anthropometric measurements were performed. A fasting blood sample was drawn on the same day as the 24 hr dietary recalls (DR). Intake of fatty acids as well as that of total energy and other nutrients was assessed using a 24 hr DR that was administered by a study nurse or a clinical officer, each with training by a registered dietitian and aided by commercial food models. Foods and amounts consumed over the preceding 24 hours were recorded by the interviewer. Food composition and nutrient quantity were computed from a modified food composition database using NDS-R software (Nutrition Data System for research software version 2006, developed by the Nutrition Coordinating Center, University of Minnesota, Minneapolis, MN, USA (http://www.ncc.umn.edu/)) [16]. The NDS-R nutrient database was supplemented with nutrient composition data for local staple foods already published by the Zambian National Food and Nutrition Commission (available from http://www.nfnc.org.zm/).

### 2.3. Laboratory Assays

Participants were asked to come to the clinic after an 8-hour fast before their blood draw. Participants who reported being in a nonfasting state were rescheduled to return after fasting. Plasma and serum specimens were collected from each participant and used to determine total cholesterol (TC), high density lipoprotein-cholesterol (HDL-C), low-density lipoprotein-cholesterol (LDL-C), triglycerides, insulin, glucose, creatinine, CRP, and albumin. Methods for metabolic assays in DGPLEAD have been described previously [6, 17].

Lipid profiles and glucose were measured using a Roche Cobas Integra 400+ autoanalyzer (Roche Diagnostics, Indianapolis, IN, USA). Triglycerides, LDL-C, and TC concentrations were measured using an enzymatic colorimetric assay while HDL-C was measured using a homogeneous enzymatic colorimetric assay. Serum creatinine, CRP, and albumin concentrations were determined on a Roche Modular P analyzer using bromocresol purple assay for albumin and immunoturbidimetric assay for CRP (Roche Diagnostics, Indianapolis, IN, USA).

Fatty acid concentrations were measured in the phospholipid fraction of plasma using gas chromatography. The extraction and quantification of fatty acids were performed at the University of Minnesota using a standard validated assay that has been described in detail [18–20]. This assay identifies 29 individual fatty acids ranging from 12:0 through 24:1n9.

### 2.4. Exposure and Dependent Variable Definitions

For the first objective, we focused on pairwise Spearman rank correlations between diet and plasma PUFA measurements. In the second objective, total plasma PUFA and SFA were the main exposure variables. The dependent variables were markers of HIV/AIDS disease progression (i.e., BMI, CD4+ cell count, and serum albumin) and cardiovascular disease risk (i.e., CRP, triglycerides, HDL-C, and LDL-C). In secondary analyses with plasma arachidonic acid as the main independent variable we investigated BMI, CD4+ cell count, plasma albumin, CRP, triglycerides, HDL-C, LDL-C, and plasma albumin as dependent variables.

### 2.5. Statistical Analysis

Of the 210 participants, 90% had complete data on fatty acids in plasma. Fatty acids in plasma were expressed as a percentage of the total fatty acids analyzed while dietary fatty acids were expressed as a percentage of total energy per day [21]. To validate fatty acid intakes, we computed pairwise Spearman correlation coefficients for each PUFA estimated from 24 hr DR against a corresponding PUFA measured in plasma. The exception was plasma *α*-linolenic acid in plasma which was tested for correlation with total dietary linolenic acid since *α*- and *γ*-linolenic acid could not be separated in our 24 hour DR.

Next we determined whether plasma fatty acids are associated with CRP and lipid profiles using multivariable linear regression models. For each of the dependent variables, that is, CRP, triglycerides, HDL-C, and LDL-C we estimated associations with the exposure variables, namely, total plasma PUFA and SFA adjusted for age, sex, BMI, plasma monounsaturated fatty acids (MUFA),* trans* fatty acid, alcohol use, and smoking.

Additional analyses were conducted to understand whether individual PUFA was associated with markers of CVD risk and HIV disease progression (e.g., CD4+ counts and plasma albumin). In this analysis, Spearman rank correlations between plasma AA and each of the markers of CVD risk and HIV disease progression (e.g., CRP, lipids, CD4+ count, albumin, and BMI) were determined. We then distributed plasma AA concentrations into quartiles and used ANOVA with robust variance estimator to determine whether markers of CVD risk and HIV/AIDS disease progression significantly varied by quartiles of AA before and after adjustment for age, sex, smoking, and alcohol consumption.

Data were analyzed using SAS version 9.4 (Cary, NC) and STATA version 12.1 (College Station, TX).

## 3. Results

### 3.1. Description of the Population

The characteristics of the study population are shown in Table 1. Most participants were women (54%) and relatively young, with a median age of 32 years for women and 35 years for men. The proportion of participants with BMI <18.5 kg/m^2^ was 32%. Although the median CD4+ cell count was somewhat higher in women (143 cells/*μ*L) compared to men (129 cells/*μ*L), this difference did not reach statistical significance (*p* > 0.05). Concentrations of CRP also tended to be higher in women than in men but the differences were not statistically significant (*p* > 0.05). The frequency of smoking was quite low in our study population with only one (0.9%) current smoker among women and 10 (11%) among men (*p* < 0.05).

### 3.2. Validation of the Fatty Acid Estimates from 24 hr DR

**Figure 1:**
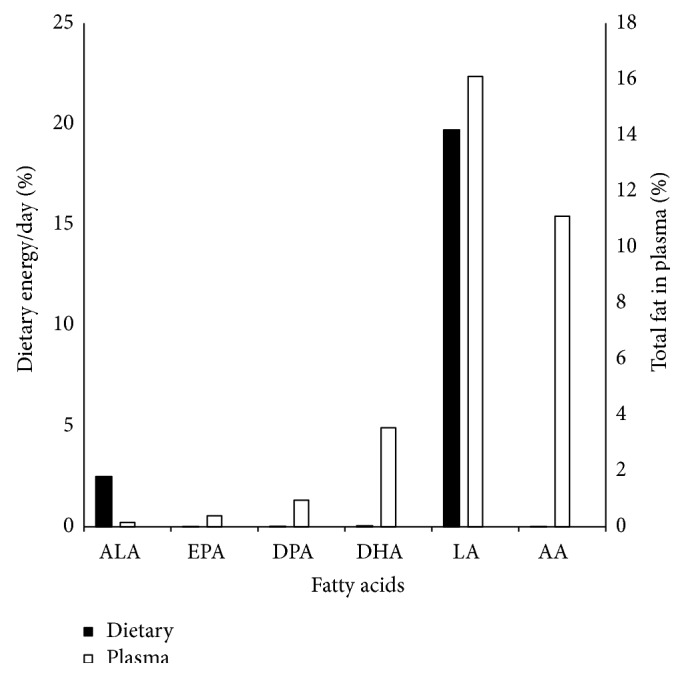
Proportions of major n-3 and n-6 fatty acids in diet and plasma of HIV/AIDS patients enrolled from January to December, 2007, in Lusaka, Zambia. Values are median intakes for 24-hour dietary recalls and median proportions for plasma fatty acids. ALA = *α*-Linolenic acid; EPA = eicosapentaenoic acid; DPA = docosapentaenoic acid; DHA = docosahexaenoic acid; LA = linoleic acid; AA = arachidonic acid.

The proportional distributions of fatty acids in the diet expressed as percent energy intake per day and in the plasma as percent of total fat are shown in Figure 1. The highest median percent energy was from linoleic acid (LA) (11.5%) and the lowest was from EPA (0.007%). The highest median percent of total fat in the plasma was from LA (16.1%) and the lowest was from *α*-linolenic acid (ALA) (0.15%).

Table 2 shows the Spearman correlation coefficients between plasma and corresponding dietary PUFA. The correlations are for the major n-3 and n-6 fatty acids. Two of the six fatty acids examined showed statistically significant correlations. The correlations ranged from very low to moderately low. The highest significant correlation was for EPA (*r* = 0.36, *p* < 0.01) and the second highest was for DHA (*r* = 0.21, *p* = 0.005). The correlations for ALA, DPA, LA, and AA were not statistically significant.

In multivariable linear regression analyses, total plasma PUFA concentrations were not significantly associated with CRP on a log scale (*β* = −0.10; 95% CI: −0.22 to 0.02, *p* = 0.09) in analyses adjusting for age, sex, BMI, MUFA,* trans* fatty acids, current smoking, and alcohol status (data not shown). A positive, though weak, association was observed between total plasma PUFA and triglycerides on a log scale (*β* = 0.03; 95% CI: 0.002 to 0.06, *p* = 0.04). No significant associations were observed between total plasma PUFA and HDL-C or LDL-C.

Plasma AA was inversely correlated with CRP (spearman correlation coefficient, *r* = −0.19, *p* = 0.02) and triglycerides (*r* = −0.14, *p* = 0.05) and positively associated with CD4+ count (*r* = 0.16, *p* = 0.02), albumin (*r* = 0.44, *p* < 0.001), HDL-C (*r* = 0.26, *p* = 0.01), and LDL-C (*r* = 0.29, *p* = 0.001). As shown in Table 3, the associations between AA and CRP, serum albumin, triglycerides, HDL-C, and LDL-C remained significant after adjustment for age, sex, smoking, and alcohol consumption in ANOVA models.

In multivariable regression, a positive association was also observed between total plasma SFA and CRP (*β* = 0.24; 95% CI: 0.08 to 0.40, *p* = 0.003) and between SFA and triglyceride concentrations (*β* = 0.08; 95% CI: 0.03 to 0.12, *p* < 0.01). We did not detect an association with HDL-C and LDL-C.

## 4. Discussion

We observed modest correlations between EPA and DHA estimated from 24 hr DR and corresponding measures in plasma but weak correlations for other PUFAs. We also observed null associations between total plasma PUFA and markers of HIV/AIDS disease progression and CVD-risk, but significant beneficial associations were observed between plasma AA and these markers. Higher plasma AA concentrations were significantly associated with higher HDL-C, LDL-C, CD4+ cell counts, and serum albumin, a profile consistent with improved patient survival.

The finding that ALA provided the highest median percent energy intake for the n-3 dietary fatty acids (1.5% or 2.5 g/day) is consistent with the notion that ALA is the major form of n-3 fatty acids obtained from dietary sources [22, 23]. The reported intake for ALA in our study is slightly higher than the range reported among western adults (0.5 to 2 g/d) [24], probably due to higher content of foods from plant sources in Zambia compared to western countries. The highest median percent proportion of total fat in the plasma among the n-3 fatty acids was from DHA (3.5%). The observation that LA contributed the highest median dietary percent energy (11.5% or 19.7 g/day) is consistent with LA's position as the major n-6 fatty acid obtained from the diet [22, 23]. As in the diet, LA was the major n-6 PUFA in plasma. Dietary LA undergoes a metabolic process which converts it to various n-6 fatty acids including AA. This may explain why even if our study reported very low median dietary intake of AA (0.04%) compared to LA (11.5%), the plasma proportions were closer (AA 11.1%, LA 16.1%).

The sources of ALA and n-6 PUFA in the Zambian diet are likely to include vegetable oils such as soybean and sunflower oil. Other sources are nuts, meats, and eggs. These food items are readily available and generally affordable in urban areas, which may explain the relatively high consumption levels reported in our study. Fish and other forms of seafood are much better sources of very long chain n-3 fatty acids such as EPA and DHA [24] but are not readily available. In Zambia, despite the availability of large bodies of fresh waters from rivers and lakes, the fish industry is not well developed; very little fish farming is practiced [15]. Thus, consumption of long-chain fatty acids in Zambia (0.08 g/day for EPA, DPA, and DHA) is lower than what is reported among adults in Northern and Eastern Europe, North America, and Australasia (mean intakes ~0.15–0.25 g/d) [25].

The correlation coefficients for EPA and DHA measured in 24 hr DR and plasma in our study were 0.36 and 0.21, respectively. These correlations were somewhat higher than those observed for EPA (*r* = 0.21) and lower than that for DHA (*r* ranges from 0.42 to 0.48), LA (*r* = 0.25), and ALA (*r* = 0.23) as measured from food frequency questionnaire and plasma studies [26, 27]. The correlations were also higher than those observed for EPA and DHA measured from a food frequency questionnaire and adipose tissue (*r* = 0.15 and 0.18, resp.) [21]. Comparable correlations of 0.15 and 0.61 for EPA and higher correlations of 0.47 and 0.57 for DHA in men and women, respectively, have been reported in a previous study [28]. In another diet-adipose tissue study, the correlation for PUFA was 0.4 and that for linolenic was 0.34 [29].

The finding from our study that total plasma PUFA was not associated with lower CRP and lipid profiles may be a result of opposing actions among fatty acids when combined as total PUFA, in contrast with the roles they play individually. For example, supplementation with fish oil rich in EPA and DHA (n-3 PUFAs) has been reported to reduce inflammation in conditions such as colitis in both animal and clinical studies [30, 31]. In contrast, AA (an n-6 PUFA) is known to exert both proinflammatory and anti-inflammatory effects through its metabolites [32, 33].

Our finding that AA was inversely associated with CRP and triglycerides and positively associated with HDL-C, CD4+ cell count and plasma albumin suggests that individual fatty acids may influence clinical outcomes in HIV/AIDS patients. However, this observation needs further investigation.

The observation that SFA was positively associated with CRP (*β* = 0.24, *p* = 0.003) is consistent with findings from a previous study in which, after adjusting for other covariates, SFA emerged as the single most important nutrient contributing to an increase in serum CRP levels [34]. The finding that SFA was positively associated with serum triglyceride concentration (*β* = 0.08, *p* < 0.01) supports evidence from previous studies that found SFA to be positively associated with coronary heart disease risk [35, 36].

In a clinical trial, daily supplementation with 1 g of n-3 fatty acids did not reduce the rate of cardiovascular events in patients at high risk for cardiovascular events [37]. However, the study reported a significant reduction in triglyceride levels (0.16 mmol/L), more among patients receiving n-3 fatty acids than among those receiving placebo (*p* < 0.001), without a significant effect on other lipids. Similarly, in our secondary analyses total n-3 fatty acids were not associated with reductions in CRP or improved lipid profiles and this may justify the need to explore the role of individual fatty acids in improving the CVD risk profiles.

The cross-sectional nature of our study limits causal inference between the exposure variables and the dependent variables of interest. The study could have been prone to interviewer bias arising from inconsistency in the way the DR was administered by different interviewers. The study could also have been prone to reporting bias which may have arisen from the participants being inclined to report healthier foods more than the less healthy foods. To mitigate these potential biases, the interviewers were specifically trained to elicit complete dietary histories from all participants. Lastly, the study population of adult HIV patients was recruited at a single health facility, which could limit generalizability to HIV-infected individuals in other settings. We also acknowledge that because the study was done among patients not yet on ART, we do not know the associations between fatty acids and markers of HIV/AIDS disease progression or CVD risk among those already on ART. Studies that assess effects of ART before and during ART are warranted so as to determine whether supplementation with fatty acids will modulate outcomes from ART in resource-limited settings.

## 5. Conclusions

The significant but generally low diet-plasma long-chain PUFA correlations could suggest that a single self-reported 24 hr DR may be inadequate for assessing PUFA intake in HIV/AIDS patients in Zambia. The study also suggests that SFA, which were positively related to markers of CVD risk, could play a role in HIV-related cardiovascular disease. Our study has found no evidence that total PUFA are inversely associated with CVD risk markers in HIV patients. However, there was evidence from secondary analyses that individual fatty acids, particularly AA, may play a role in improving CVD risk profiles and markers of HIV/AIDS disease progression.

## Figures and Tables

**Table 1:** Baseline characteristics of the study population.

Variable	Women *n* = 113	Men *n* = 97	*p*
Age, years	32 (27, 37)	35 (31, 40)	0.003
BMI, kg/m^2^	19.6 (18.0, 21.7)	19.6 (18.0, 21.3)	0.62
Current smoker, *n* (%)	1 (0.93)	10 (10.5)	0.003
Current drinker, *n* (%)	10 (9.26)	12 (12.6)	0.41
CD4, count, cell/*µ*L	143 (108, 175)	129 (88, 168)	0.07
C-reactive protein, mg/L	12.6 (2.01, 32.2)	5.71 (1.51, 32.1)	0.24
Albumin, g/dL	3.00 (2.60, 3.40)	3.20 (2.60, 3.70)	0.09
Total cholesterol, mmol/L	3.56 (2.94, 4.11)	3.21 (2.68, 3.75)	0.01
Triglycerides, mmol/L	1.10 (0.89, 1.51)	1.05 (0.81, 1.43)	0.39
LDL-cholesterol, mmol/L	2.13 (1.60, 2.50)	1.77 (1.34, 2.38)	0.02
HDL-cholesterol, mmol/L	0.81 (0.47, 1.09)	0.71 (0.52, 1.04)	0.42
TC : HDL-C, ratio	4.49 (3.44, 7.00)	4.73 (3.32, 6.12)	0.47
Total energy intake, kcal/day	1588 (1179, 1956)	1777 (1356, 2305)	0.03
Total fat, % energy/day	34.1 (27.8, 42.2)	28.9 (20.5, 35.5)	<0.0001
Total *trans* fatty acids, % energy/day	0.27 (0.09, 0.51)	0.30 (0.12, 0.63)	0.37
Total saturated fatty acids, % energy/day	6.52 (5.12, 8.00)	5.52 (3.74, 7.64)	0.02
Total MUFA, % energy/day	9.47 (7.84, 11.8)	8.05 (5.72, 10.9)	0.01
Total dPUFA, % energy/day	15.1 (11.4, 19.2)	11.1 (8.0, 14.8)	<0.0001
n-3 dPUFAs, % energy/day	1.89 (1.30, 2.60)	1.40 (0.92, 1.99)	<0.0001
n-6 dPUFAs, % energy/day	14.6 (10.9, 18.6)	10.9 (7.8, 14.0)	<0.0001
Total pPUFA, % total fat	37.1 (35.2, 39.1)	36.2 (34.4, 38.4)	0.07
n-3 pPUFA, % total fat	5.51 (4.57, 5.98)	4.85 (4.28, 5.49)	0.01
n-6 pPUFA, % total fat	31.9 (29.9, 33.1)	31.7 (29.6, 33.3)	0.38

Values are median (25th, 75th percentile) unless otherwise stated.

BMI, body mass index; HDL, high density lipoprotein; LDL, low density lipoprotein; MUFA, monounsaturated fatty acids; PUFA, polyunsaturated fatty acids; dPUFA, PUFA from dietary sources; pPUFA, PUFA levels determined from plasma; TC, total cholesterol.

**Table 2:** Spearman correlation coefficients between plasma and dietary polyunsaturated fatty acids.

PUFA type	Plasma composition (% of total fat)	Dietary composition (% energy/day)	*r*	*p*
ALA (18:3n-3)	0.15 (0.12, 0.20)	1.50 (0.96, 1.92)	−0.05	0.50
EPA (20:5n-3)	0.39 (0.28, 0.51)	0.01 (0, 0.12)	0.36	<0.01
DPA (22:5n-3)	0.95 (0.77, 1.13)	0.01 (0, 0.03)	−0.01	0.90
DHA (22:6n-3)	3.54 (2.98, 4.27)	0.03 (0, 0.17)	0.21	0.01
LA (18:2n-6)	16.1 (14.6, 18.0)	11.5 (8.10, 14.9)	0.06	0.09
AA (20:4n-6)	11.1 (9.58, 12.3)	0.04 (0.01, 0.08)	0.09	0.21

Plasma and dietary composition values are median (25th, 75th percentile).

AA, arachidonic acid; ALA, *α*-linolenic acid; DHA, docosahexaenoic acid; DPA, docosapentaenoic acid; EPA, eicosapentaenoic acid; LA, linoleic acid; PUFA, polyunsaturated fatty acid.

**Table 3:** Quartiles of plasma arachidonic acid (AA) levels and markers of cardiovascular disease and HIV/AIDS progression.

Variable	Model	1st Quartile (*n* = 49)	2nd (*n* = 49)	3rd (*n* = 49)	4th (*n* = 49)	*p*
BMI, kg/m^2^	1	19.6 ± 0.34	19.9 ± 0.37	20.3 ± 0.41	20.5 ± 0.46	0.38
	2	20.1 ± 0.5	20.5 ± 0.5	20.8 ± 0.6	20.9 ± 0.6	0.39

CD4, cells/*μ*L	1	130 ± 9	122 ± 8	145 ± 7	147 ± 7	0.02
	2	133 ± 12	126 ± 13	145 ± 12	150 ± 13	0.04

Albumin, g/dL	1	2.6 ± 0.1	3.1 ± 0.1	3.2 ± 0.1	3.4 ± 0.1	<0.0001
	2	2.7 ± 0.1	3.2 ± 0.2	3.3 ± 0.1	3.5 ± 0.2	<0.0001

C-reactive protein, mg/L	1	32.2 ± 8.1	25.6 ± 6.7	30.2 ± 7.2	11.4 ± 2.4	0.004
	2	26.9 ± 8.2	19.9 ± 6.5	24.8 ± 7.5	4.3 ± 5.6	0.01

Triglycerides, mmol/L	1	1.30 ± 0.09	1.27 ± 0.10	1.37 ± 0.09	1.04 ± 0.06	0.01
	2	1.26 ± 0.15	1.24 ± 0.16	1.37 ± 0.19	0.98 ± 0.15	0.003

HDL-C, mmol/L	1	0.61 ± 0.06	0.81 ± 0.07	0.79 ± 0.06	0.99 ± 0.09	0.004
	2	0.68 ± 0.09	0.86 ± 0.11	0.84 ± 0.09	1.05 ± 0.12	0.01

LDL-C, mmol/L	1	1.66 ± 0.13	1.99 ± 0.10	2.17 ± 0.09	2.12 ± 0.08	0.01
	2	1.78 ± 0.18	2.12 ± 0.15	2.24 ± 0.14	2.22 ± 0.15	0.02

Model 1: unadjusted; Model 2: means and standard errors are adjusted for age, sex, and smoking and alcohol consumption. *p*-values are from ANOVA models that use robust standard errors.

## References

[1] UNAIDS (2010). *Report on the Global AIDS Epidemic 2010, Joint United Nations Programme on HIV/AIDS*.

[2] Koethe J. R., Lukusa A., Giganti M. J. (2010). Association between weight gain and clinical outcomes among malnourished adults initiating antiretroviral therapy in Lusaka, Zambia. *Journal of Acquired Immune Deficiency Syndromes*.

[3] Stringer J. S. A., Zulu I., Levy J. (2006). Rapid scale-up of antiretroviral therapy at primary care sites in Zambia: feasibility and early outcomes. *Journal of the American Medical Association*.

[4] Heimburger D. C., Koethe J. R., Nyirenda C. (2010). Serum phosphate predicts early mortality in adults starting antiretroviral therapy in Lusaka, Zambia: a prospective cohort study. *PLoS ONE*.

[5] Müller M., Wandel S., Colebunders R., Attia S., Furrer H., Egger M. (2010). Immune reconstitution inflammatory syndrome in patients starting antiretroviral therapy for HIV infection: a systematic review and meta-analysis. *The Lancet Infectious Diseases*.

[6] Ngu J. N., Heimburger D. C., Arnett D. K. (2010). Fasting triglyceride concentrations are associated with early mortality following antiretroviral therapy in Zambia. *North American Journal of Medical Sciences*.

[7] Carrol D. N., Roth M. T. (2002). Evidence for the cardioprotective effects of omega-3 fatty acids. *Annals of Pharmacotherapy*.

[8] Djoussé L., Hunt S. C., Arnett D. K., Province M. A., Eckfeldt J. H., Ellison R. C. (2003). Dietary linolenic acid is inversely associated with plasma triacylglycerol: the National Heart, Lung, and Blood Institute Family Heart Study. *American Journal of Clinical Nutrition*.

[9] Djoussé L., Folsom A. R., Province M. A., Hunt S. C., Ellison R. C. (2003). Dietary linolenic acid and carotid atherosclerosis: the National Heart, Lung, and Blood Institute Family Heart Study. *The American Journal of Clinical Nutrition*.

[10] Okamoto Y., Okano K., Izuishi K., Usuki H., Wakabayashi H., Suzuki Y. (2009). Attenuation of the systemic inflammatory response and infectious complications after gastrectomy with preoperative oral arginine and *ω*-3 fatty acids supplemented immunonutrition. *World Journal of Surgery*.

[11] Reinders I., Virtanen J. K., Brouwer I. A., Tuomainen T.-P. (2012). Association of serum n-3 polyunsaturated fatty acids with C-reactive protein in men. *European Journal of Clinical Nutrition*.

[12] Gerber J. G., Kitch D. W., Fichtenbaum C. J. (2008). Fish oil and fenofibrate for the treatment of hypertriglyceridemia in HIV-infected subjects on antiretroviral therapy: results of ACTG A5186. *Journal of Acquired Immune Deficiency Syndromes*.

[13] Siamusantu W. S. (2009). *Zambia Nutrition Profile—Nutrition and Consumer Protection Division*.

[14] Reddy K. S. (2001). *Chronic Disease Epidemics in Developing Countries: Can We Telescope Transition?*.

[15] Nyirenda D. B., Musukwa M., Mugode R. H., Shindano J. (2005). *Revised Food Composition Tables of Zambia*.

[16] Koethe J. R., Blevins M., Bosire C. (2013). Self-reported dietary intake and appetite predict early treatment outcome among low-BMI adults initiating HIV treatment in sub-Saharan Africa. *Public Health Nutrition*.

[17] Kiage J. N., Heimburger D. C., Nyirenda C. K. (2013). Cardiometabolic risk factors among HIV patients on antiretroviral therapy. *Lipids in Health and Disease*.

[18] Kabagambe E. K., Tsai M. Y., Hopkins P. N. (2008). Erythrocyte fatty acid composition and the metabolic syndrome: a National Heart, Lung, and Blood Institute GOLDN study. *Clinical Chemistry*.

[19] Kabagambe E. K., Ordovas J. M., Hopkins P. N., Tsai M. Y., Arnett D. K. (2012). The relation between erythrocyte trans fat and triglyceride, VLDL- and HDL-cholesterol concentrations depends on polyunsaturated fat. *PLoS ONE*.

[20] Cao J., Schwichtenberg K. A., Hanson N. Q., Tsai M. Y. (2006). Incorporation and clearance of omega-3 fatty acids in erythrocyte membranes and plasma phospholipids. *Clinical Chemistry*.

[21] Baylin A., Kabagambe E. K., Siles X., Campos H. (2002). Adipose tissue biomarkers of fatty acid intake. *American Journal of Clinical Nutrition*.

[22] Kris-Etherton P. M., Taylor D. S., Yu-Poth S. (2000). Polyunsaturated fatty acids in the food chain in the United States. *American Journal of Clinical Nutrition*.

[23] DHA/EPA Omega 3-Institute (2013). *Metabolism of Omega-6 and Omega-3 Fatty Acids and the Omega 6: Omega 3 Ratio*.

[24] British Nutrition Foundation (1999). *Briefing Paper: n-3 Fatty Acids and Health*.

[25] Scientific Advisory Committee on Nutrition (SACN) (2004). *Advice on Fish Consumption: Benefits and Risks*.

[26] Sun Q., Ma J., Campos H., Hankinson S. E., Hu F. B. (2007). Comparison between plasma and erythrocyte fatty acid content as biomarkers of fatty acid intake in US women. *The American Journal of Clinical Nutrition*.

[27] Berstad P., Thiis-Evensen E., Vatn M. H., Almendingen K. (2012). Fatty acids in habitual diet, plasma phospholipids, and tumour and normal colonic biopsies in young colorectal cancer patients. *Journal of Oncology*.

[28] Tjonneland A., Overvad K., Thorling E., Ewertz M. (1993). Adipose tissue fatty acids as biomarkers of dietary exposure in Danish men and women. *American Journal of Clinical Nutrition*.

[29] Garland M., Sacks F. M., Colditz G. A. (1998). The relation between dietary intake and adipose tissue composition of selected fatty acids in US women. *American Journal of Clinical Nutrition*.

[30] Camuesco D., Gálvez J., Nieto A. (2005). Dietary olive oil supplemented with fish oil, rich in EPA and DHA (n-3) polyunsaturated fatty acids, attenuates colonic inflammation in rats with DSS-induced colitis. *The Journal of Nutrition*.

[31] Lorenz R., Weber P. C., Szimnau P., Heldwein W., Strasser T., Loeschke K. (1989). Supplementation with n-3 fatty acids from fish oil in chronic inflammatory bowel disease—a randomized, placebo-controlled, double-blind cross-over trial. *Journal of Internal Medicine*.

[32] Ricciotti E., FitzGerald G. A. (2011). Prostaglandins and inflammation. *Arteriosclerosis, Thrombosis, and Vascular Biology*.

[33] Serhan C. N., Krishnamoorthy S., Recchiuti A., Chiang N. (2011). Novel anti-inflammatory—pro-resolving mediators and their receptors. *Current Topics in Medicinal Chemistry*.

[34] Arya S., Isharwal S., Misra A. (2006). C-reactive protein and dietary nutrients in urban Asian Indian adolescents and young adults. *Nutrition*.

[35] Heimburger D. C., Ard J. D. (2006). *Handbook of Clinical Nutrition*.

[36] Khaw K. T., Friesen M. D., Riboli E., Luben R., Wareham N. (2012). Plasma phospholipid fatty acid concentration and incident coronary heart disease in men and women: the EPIC-Norfolk prospective study. *PLoS Medicine*.

[37] Bosch J., Gerstein H. C., Dagenais G. R. (2012). n-3 fatty acids and cardiovascular outcomes in patients with dysglycemia. *The New England Journal of Medicine*.

